# The Effects of Transcranial Direct Current Stimulation (tDCS) on Working Memory Training in Healthy Young Adults

**DOI:** 10.3389/fnhum.2019.00019

**Published:** 2019-02-01

**Authors:** Yufeng Ke, Ningci Wang, Jiale Du, Linghan Kong, Shuang Liu, Minpeng Xu, Xingwei An, Dong Ming

**Affiliations:** ^1^Academy of Medical Engineering and Translational Medicine, Tianjin University, Tianjin, China; ^2^Department of Biomedical Engineering, College of Precision Instruments and Optoelectronics Engineering, Tianjin University, Tianjin, China

**Keywords:** transcranial direct current stimulation (tDCS), working memory, working memory training, cognitive enhancement, cognitive training

## Abstract

Working memory (WM) is a fundamental cognitive ability to support complex thought, but it is limited in capacity. WM training has shown the potential benefit for those in need of a higher WM ability. Many studies have shown the potential of transcranial direct current stimulation (tDCS) to transiently enhance WM performance by delivering a low current to the brain cortex of interest, *via* electrodes on the scalp. tDCS has also been revealed as a promising intervention to augment WM training in a few studies. However, those few tDCS-paired WM training studies, focused more on the effect of tDCS on WM enhancement and its transferability after training and paid less attention to the variation of cognitive performance during the training procedure. The current study attempted to explore the effect of tDCS on the variation of performance, during WM training, in healthy young adults. All the participants received WM training with the load-adaptive verbal N-back task, for 5 days. During the training procedure, active/sham anodal high-definition tDCS (HD-tDCS) was used to stimulate the left dorsolateral prefrontal cortex (DLPFC). To examine the training effect, pre- and post-tests were performed, respectively, 1 day before and after the training sessions. At the beginning of each training session, stable-load WM tasks were performed, to examine the performance variation during training. Compared to the sham stimulation, higher learning rates of performance metrics during the training procedure were found when WM training was combined with active anodal HD-tDCS. The performance improvements (post–pre) of the active group, were also found to be higher than those of the sham group and were transferred to a similar untrained WM task. Further analysis revealed a negative relationship between the training improvements and the baseline performance. These findings show the potential that tDCS may be leveraged as an intervention to facilitate WM training, for those in need of a higher WM ability.

## Introduction

Cognitive training, like working memory (WM) training, has shown the potential to produce broad benefits for those who have special requirements in their cognitive abilities, or for those who suffer from cognitive impairments (Richmond et al., 2014; Au et al., 2016; Choe et al., 2016; Stephens and Berryhill, 2016; Ciechanski and Kirton, 2017; Ruf et al., 2017; Talsma et al., 2017). As a fundamental and essential cognitive ability, WM supports complex thought but is limited in capacity. Thus, WM training interventions have become popular as a means of potentially improving WM-related cognitive abilities for those in need (Au et al., 2016). Transcranial direct current stimulation (tDCS), which has shown potential to modulate brain cortical excitability and activity, by transmitting a weak electric current into the brain (Andrews et al., 2011; Coffman et al., 2014; Santarnecchi et al., 2015), has been found as a possible way to improve WM (Dockery et al., 2011; Brunoni and Vanderhasselt, 2014; Park et al., 2014; Richmond et al., 2014), sustained attention (Nelson et al., 2014), motor learning (Ciechanski and Kirton, 2017), multitasking (Filmer et al., 2013; Hsu et al., 2015; Nelson et al., 2016) and so on. Cognitive enhancement, using tDCS, has therefore attracted increased attention over the last decade.

A considerable quantity of single-session studies using tDCS have revealed potential benefits in improving participants’ performance in WM tasks. In one particularly noteworthy study, carried out by Fregni et al. (2005), anodal tDCS (a-tDCS) applied to the left dorsolateral prefrontal cortex (DLPFC), increased response accuracy of the WM task performed concurrently with the stimulation. However, no significant effect appeared when they applied anodal stimulation over the primary motor cortex and cathodal stimulation over the left DLPFC. These findings indicate that the enhancing effect of tDCS on WM memory depends on the stimulation polarity and is specific to the site of stimulation (Fregni et al., 2005). Many subsequent studies compared factors like electrode placement, current density and stimulation duration that may affect the efficacy of tDCS and found that the anodal stimulation of the left prefrontal, tended to enhance WM performance (Coffman et al., 2014; Richmond et al., 2014; Moreno et al., 2015; Santarnecchi et al., 2015; Au et al., 2016; Gözenman and Berryhill, 2016; Hill et al., 2016a, 2017; Stephens and Berryhill, 2016; Trumbo et al., 2016; Ruf et al., 2017; Talsma et al., 2017). Neuroimaging studies using EEG and functional near-infrared spectroscopy (fNIRS) have provided evidence that tDCS can alter brain activities (McKendrick et al., 2015; Bergmann et al., 2016; Wörsching et al., 2016; Jones et al., 2017; Bogaard et al., 2019). In addition to WM related studies, tDCS has also shown potential to mitigate vigilance decrement (Nelson et al., 2014) and enhance multitasking performance (Nelson et al., 2016).

Though many published studies have provided positive results, there has also been a debate over the efficacy of tDCS on human’s cognitive functions for healthy and neuropsychiatric cohorts. Hill et al. (2016a) systematically reviewed and meta-analyzed the (single-session and multi-session) studies on a-tDCS of DLPFC + WM, published between July 1998 and June 2014, and found that a-tDCS tended to improve offline (shortly after a-tDCS) WM performance in healthy cohorts and online (during a-tDCS) WM performance in neuropsychiatric cohorts. However, another quantitative review found no evidence of the cognitive effects in healthy populations from single-session tDCS for executive function, language, memory, and miscellaneous tasks (Horvath et al., 2015). A subsequent meta-analysis of the effect of a-tDCS on WM in healthy populations, found that left DLPFC stimulation alone had no significance after publication bias correction, but that left DLPFC stimulation coupled with WM training had a significant effect (Mancuso et al., 2016). With a more stringent publication bias correction method *p-curve* (Simonsohn et al., 2014), Medina and Cason further analyzed the studies in Mancuso et al. (2016) and concluded that the tDCS studies had no evidential value (Medina and Cason, 2017). By summarizing these reviews, the efficacy of tDCS for WM seems to remain somewhat uncertain, especially single-session tDCS studies. This however, does not mean that these reviews can provide definitive evidence for the ineffectiveness of tDCS for cognitive processes. As Medina and Cason said, the stimulation parameters (like stimulation site, polarity, current, reference electrode location, length of stimulation, when stimulation occurred, and other factors) varied in the studies they reviewed which may affect the results of the meta-analysis (Medina and Cason, 2017). Individual differences, like baseline ability, education level and genetic factors, were also found to be important factors that may affect the efficacy of tDCS but has not received sufficient attention (Moreno et al., 2015; Gözenman and Berryhill, 2016; Hill et al., 2016a; Katz et al., 2017; Stephens et al., 2017; Talsma et al., 2017). Another point that should be noted, is that the above reviews mainly focused on studies that utilized tDCS alone in single-session protocols, as an insufficient number of multi-session studies were published before 2014. According to the limited number of publications, the WM enhancement potential of tDCS was found to most probably lie in its use during training (Mancuso et al., 2016).

Based on the assumption that tDCS has the potential to modulate neuronal excitability and synaptic plasticity (Hummel and Cohen, 2006; Santarnecchi et al., 2015), a number of studies have explored the effect of tDCS on cognitive training in the last 3 years. A study in a non-human primate model found that tDCS, coupled with multi-session learning, facilitated associative learning and altered functional connectivity by analyzing the behavioral outcomes and local field potential (Krause et al., 2017). In a three-session WM training study, implemented in healthy adults, the advantage of WM training combined with a-tDCS was not only presented immediately after the training, but also in the follow-up session up to 9 months after the training (Ruf et al., 2017). A-tDCS related benefits maintained stable even as long as a year after the original intervention, in a 7-day tDCS-paired WM training study in young healthy adults (Au et al., 2016; Katz et al., 2017). The enhancement of cortical efficiency and connectivity was also demonstrated in a study which found a significant improvement of WM ability through a-tDCS paired WM training in young healthy adults (Jones et al., 2017). Studies in older healthy adults also found that a-tDCS paired WM training induced significantly greater improvements 1 month after the training (Jones et al., 2015; Stephens and Berryhill, 2016). In the study conducted by Richmond et al. (2014), they revealed that tDCS did not change the rate of learning over time, but shifted the entire learning curve upwards. However, in the study implemented by Ruf et al. (2017), the learning curve was steeper when WM training was combined with tDCS. In patients with fibromyalgia, a-tDCS paired WM training significantly increased immediate memory when compared to a sham (dos Santos et al., 2018). The improvements of tDCS paired multi-session WM training were also found to be transferred to untrained WM tasks (Richmond et al., 2014; Au et al., 2016; Stephens and Berryhill, 2016; Trumbo et al., 2016; Ruf et al., 2017). A recent study in monkeys provided evidence that single neuron firing rates and network interactions could be modulated by polarity and a dose of tDCS and higher a-tDCS intensity induced higher firing rates of regular firing neurons (Bogaard et al., 2019). Although a few reviews questioned the efficacy of tDCS, these recently published studies provide more evidence that WM training paired with tDCS can augment cognition.

Some reports also mentioned that the electrode montage could probably be a factor that influences the efficacy of tDCS and pointed out that the so-called high-definition tDCS (HD-tDCS), may more effectively modulate brain functions (Horvath et al., 2015; Mancuso et al., 2016). However, The majority of existing studies delivered stimulation using bipolar tDCS (BP-tDCS) montage with the stimulation electrode over the target brain regions and the return electrode placed elsewhere on or near the head (Fregni et al., 2005; Coffman et al., 2014). Computing modeling studies indicated that HD-tDCS, particularly with a central “active” electrode placed over the target brain region and four “return” electrodes surrounding it (Datta et al., 2009; Alam et al., 2016), can deliver a current to the target cortical structure with higher spatial precision and less diffuse current flow (Datta et al., 2009; Edwards et al., 2013; Saturnino et al., 2015; Alam et al., 2016; Laakso et al., 2016). A study carried out by Hill et al. (2017) compared the neurophysiological effects of BP-tDCS and HD-tDCS and revealed widespread neuromodulatory changes after 5 and 30 min HD-tDCS, but not BP-tDCS. Another study revealed that HD-tDCS showed more benefits on the WM performance of low WM capacity participants, when compared with BP-tDCS (Gözenman and Berryhill, 2016). These findings portend that HD-tDCS may potentially be more effective in modulating brain activities and behavior outcomes than BP-tDCS.

Although a few studies explored the effect of tDCS on cognitive training, those studies focused more on the effect of tDCS on cognitive enhancement after training and paid less attention to the variation of cognitive performance during the training procedure. The current study aimed to explore the effect of anodal HD-tDCS on the training procedure, by analyzing the variation of performance of the stable-load task during a multi-session load-adaptive WM training. We hypothesized that anodal HD-tDCS would facilitate the WM training procedure and boost the training speed of the stable-load task.

## Materials and Methods

### Participants

A total of 30 college students (18 males; aged 20–25), without a self-reported history of mental or neurological illness and drug abuse, volunteered to participate in this experiment and provided informed consent. All participants had normal or corrected to normal vision. All participants were evenly assigned to an active or sham tDCS group, *via* a simple random assignment. Fifteen participants received active tDCS and the other fifteen received a sham stimulation. There were no differences in age, years of education received or in pretest scores between groups.

This study was carried out in accordance with the recommendations of the institutional review board of Tianjin University, the ethics committee of the Academy of Medical Engineering and Translational Medicine at Tianjin University. All subjects gave written informed consent in accordance with the Declaration of Helsinki. The study protocol was approved by the ethics committee of the Academy of Medical Engineering and Translational Medicine, Tianjin University.

### Working Memory Task

**Figure 1:**
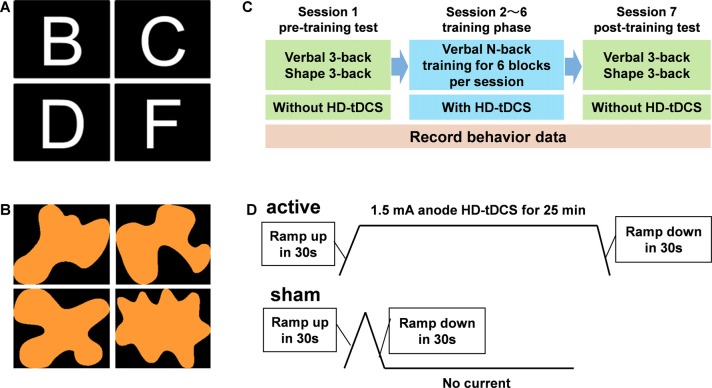
Panels **(A,B)** indicate the typical examples for the letters and shapes used respectively in verbal and shape n-back. Panel **(C)** shows the procedure of the whole experiment. Panel **(D)** indicates the variation of electric current for the active and sham group during one session.

Verbal and shape n-back tasks implemented in PsychoPy (Peirce, 2007) served as the WM task in this study. Letters in the Arial font in white color, of the 10 consonants (B, C, D, F, G, H, J, K, L and M) or shapes from the 10 abstract irregular shapes, like the ones shown in Figures 1A,B, were randomly presented in the center of the black background screen which was placed in front of the subjects, respectively, in verbal and shape n-back tasks. The size of the letters was set to 0.5 by the “letter height” option in PsychoPy. The size of the shapes was similar to the letters. Each trial lasted for 3 s, a letter or a shape was presented for 0.5 s followed by a “+” in the center of the screen for 2.5 s. The subjects were instructed to press the left/right arrow button on a keyboard when the letter or shape matched/mismatched the one n-th before, during the onset of the current one and after the onset of the next one. The match proportion was 50% for all the n-back tasks used in this study.

### Experiment Procedure

As shown in Figure 1C, every one of the volunteers received seven sessions of experiments on seven consecutive days. On the 1st day, volunteers received a pre-training test after a short training session which aimed to ensure that they were familiar enough with verbal and shape n-back tasks and that they could achieve accuracies of ~80% in verbal and shape 3-back. In the pre-training test session, four blocks of verbal 3-back and four blocks of shape 3-back were performed randomly, with 50 trials (150 s = 50 trials × 3 s/trial) per block. On the next five consecutive days, five WM training sessions followed. Each WM training session consisted of six blocks of verbal n-back tasks, a 3-back, a 4-back and four blocks of load-adaptive n-back tasks. Each block of the training session consisted of 80 trials and lasted for 4 min (240 s = 80 trials × 3 s/trial). That means the tasks in each session took 24 min (6 blocks × 4 min/block). Taking into account the breaks between two consecutive blocks, each training session lasted for about 30 min.

The load-adaptive n-back task in this study, means that the load factor *n* of the current block was adjusted according to the performance of the last block. In order to increase the difficulty of the training task, the load factor *n* of the current block would increase by one if the response accuracy of the last block was better than 85%. Otherwise, the load factor *n* would be the same with the last block. On all subsequent training days, the starting load factor *n* was determined by each individual’s performance at the end of the prior session. There was no specified cap on the load factor *n*, and the maximum that they could reach in the fifth training session depended on the training effect. The post-training test took place the day after the last training session. The tasks in the post-training test were identical to that of the pre-training test session. The reason why the shape 3-back was used in the pre- and post-training sessions, but not in the training sessions, was that the shape 3-back task served as a validation test to examine the training effect and the near-transfer effect. It should be noted that the five WM training sessions were carried out along with tDCS, while the pre- and post-training test were conducted without tDCS.

### High Definition Transcranial Direct Current Stimulation

Sham or active HD-tDCS was administered to the left DLPFC with the Starstim system (Neuroelectrics, Barcelona, Spain) *via* circular saline-soaked sponge electrodes (2.5 cm in diameter) for 25 min from the beginning of the training sessions. The HD-tDCS anode was placed in F3 and four cathodes were placed in Fp1, Fz, C3 and FT7, according to the 10-10 standard EEG system with Neuroelectrics Cap. With this montage, the central electrode (F3) was right above left DLPFC and the maximal intensity was also located in the left DLPFC. Impedance values were all verified to be <10 kΩ for the duration of the entire session. For active HD-tDCS, the current was 1.5 mA and maintained for 25 min with a ramp up/down of 30 s. For sham HD-tDCS, as shown in Figure 1D, the current was 0 mA with a ramp up and a ramp down of 30 s at the beginning of the stimulation. The settings of sham stimulation helped to mask sham and active conditions.

### Performance Metrics

WM performance metrics like response time (RT), response accuracy (ACC), and signal detection [*d’* = z(hit rate) − z(false alarm rate)] (Macmillan and Creelman, 2004) of all the tasks used in this study were recorded for further analysis. For the pre- and post-training sessions, the changes of these metrics were analyzed to examine the effect of HD-tDCS on training effects, by comparing between active and sham groups. For the training sessions, the regression lines between the performance metrics and the number of training sessions were regarded as the learning curves. The learning rates (the slopes of the learning curves) of these metrics were compared between active and sham groups, to examine the effect of HD-tDCS on the training procedure. The changes (post–pre) and the learning rates were compared between the active and sham groups.

### Statistics

One-thousand iterations bootstrapping-based, non-parametric unpaired *T*-tests were employed as comparisons because a bootstrapping based *T*-test is distribution-independent and more applicable to small sample sizes than parametric *T*-tests are (Hesterberg et al., 2003). The significance level was corrected with the false discovery rate (FDR) method (Benjamini and Yekutieli, 2001) when multiple comparisons were performed. The statistical threshold was set to *p* < 0.05, with FDR corrected for multiple comparisons [*q*_(FDR)_ < 0.05]. The FDR corrected significance level *q*_(FDR)_ and the *t*-value of the unpaired *T*-test were reported for each comparison. Statistical power analyses were performed with the online tool WebPower^1^ for all the comparisons of significance and statistical powers were reported. Linear regression between the baseline performance metrics and the corresponding learning rates and training gains, were conducted to explore the effect of baseline performance on WM training. The significance level p, the coefficient of determination *r*^2^ and the Pearson correlation coefficient *R*, were reported for the regression analyses.

## Results

### The Effect of HD-tDCS on the Learning Rates

**Figure 2:**
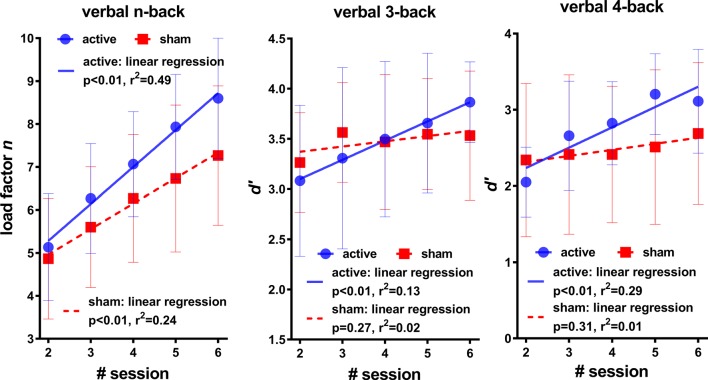
The mean values (across participants) of *n* (the highest load factor the participants could reach in the corresponding session) and *d’* and their linear regression with the session number across the training sessions.

To explore the effect of HD-tDCS on the training procedure, the variations of *n* and *d’* across the training sessions were first compared between the active and sham group. As shown in Figure 2, the left panel indicates the learning curves of the mean value of the load factor *n* that the participants could reach in each training session. It is obvious that the learning curve of the active group (slope: 0.86) tended to be steeper than that of the sham group (slope: 0.59). A group-level comparison of the slopes of learning curves of *n* found significant difference between the active group (0.86 ± 0.19) and the sham group (0.59 ± 0.17; *p* < 0.01, *t*_(28)_ = 4.06, power = 0.94). The mean values of *d’* of the active group also tended to increase with the increase of training times, for both the verbal 3- and 4-back tasks. In comparison, there seems to be no apparent increase for the mean values of *d’* of the sham group in both the verbal 3- and 4-back tasks. Linear regressions between *d’* and the training times were further applied to explore the training effect. The results, as shown in Figure 2, indicate significant linear relationships between *d’* and the training times of the active group for verbal 3-back (*p* < 0.01, *r*^2^ = 0.13) and 4-back (*p* < 0.01, *r*^2^ = 0.29). However, no significant linear relationship between *d’* and training times was found for the sham group for verbal 3-back (*p* = 0.27, *r*^2^ = 0.02) and 4-back (*p* = 0.31, *r*^2^ = 0.01).

**Figure 3:**
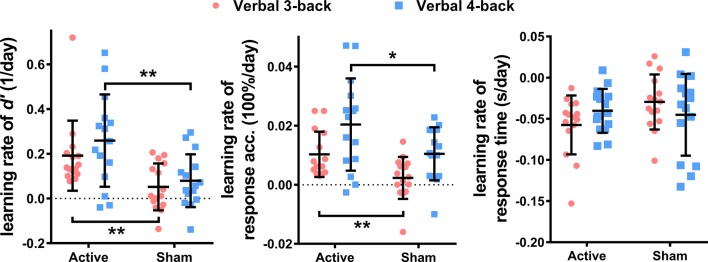
The comparisons of learning rates of *d’*, ACC, and response time (RT) between active and sham groups for verbal 3- and 4-back during the training sessions (*p < 0.05; **p < 0.01).

To further investigate the effect of HD-tDCS, the learning rates of the performance metrics were compared between the active and sham groups, with the bootstrapping based independent sample *T*-tests. The change rates of the performance metrics, during training, served as learning rates and were obtained by calculating the slopes of linear regressions between the metrics and the number of training sessions. The results, as shown in Figure 3, suggest that the active group had apparent higher learning rates than the sham group, for the performance metrics of both verbal 3- and 4-back. The statistical results showed that the active group had significantly higher learning rates for *d’* (verbal 3-back: *q*_(FDR)_ < 0.01, *t*_(28)_ = 2.86, power = 0.80; verbal 4-back: *q*_(FDR)_ < 0.01, *t*_(28)_ = 2.92, power = 0.81) and ACC (verbal 3-back: *q*_(FDR)_ < 0.01, *t*_(28)_ = 2.95, power = 0.82; verbal 4-back: *q*_(FDR)_ < 0.05, *t*_(28)_ = 2.13, power = 0.62) than the sham group, but no significant difference was found for the learning rate of RT between the active and sham group in both tasks (verbal 3-back: *q*_(FDR)_ > 0.05, *t*_(28)_ = −2.21; verbal 4-back: *q*_(FDR)_ > 0.05, *t*_(28)_ = 0.33).

### The Effect of HD-tDCS on the Changes (Post–Pre) of Performance

**Figure 4:**
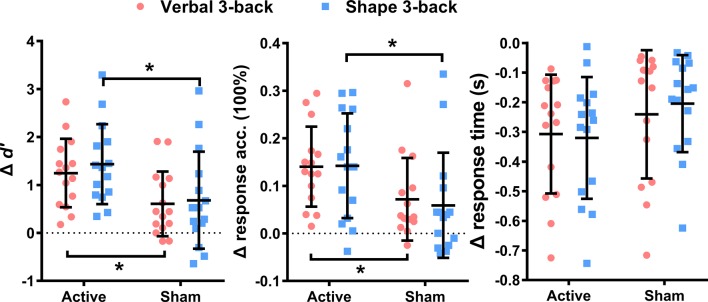
Comparisons of the training gains (post–pre) of *d’*, ACC, and RT between active and sham groups for verbal and shape 3-back tasks (*p < 0.05).

To examine the effect of HD-tDCS on the training gains of WM performance metrics, comparisons of the changes of RT, ACC, and *d’* between pre-training and post-training sessions (post–pre) were performed, between the active and sham groups, for both verbal and shape 3-back. The results, as shown in Figure 4, showed that the gains of the performance metrics of the active group tend to be higher than those of the sham group. The bootstrapping based independent sample *T*-tests found significant differences in Δ *d*’ and Δ ACC between the active and sham groups, for both verbal 3-back (*d*’: *q*_(FDR)_ < 0.05, *t*_(28)_ = 2.53, power = 0.73; ACC: *q*_(FDR)_ < 0.05, *t*_(28)_ = 2.20, power = 0.64) and shape 3-back (*d*’: *q*_(FDR)_ < 0.05, *t*_(28)_ = 2.23, power = 0.65; ACC: *q*_(FDR)_ < 0.05, *t*_(28)_ = 2.07, power = 0.60). However, the reductions in RTs (Δ RT) of the active group were not found to be significantly different from those of the sham group, for both verbal (*q*_(FDR)_ = 0.389, *t*_(28)_ = −0.88) and shape (*q*_(FDR)_ = 0.10, *t*_(28)_ = −1.71) 3-back.

### Effects of Baseline Performance on Training Outcomes

**Figure 5:**
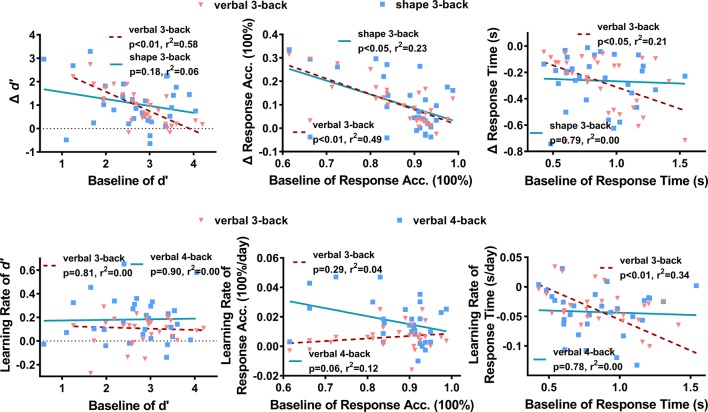
The relationships between the baseline performance metrics and the training gains (top panels) and learning rates (bottom panels).

To explore the effect of baseline performance on the learning rates and performance changes, relationships between the baseline (pre-training) performances and the corresponding learning rates and changes were analyzed with linear regression. The results, as shown in Figure 5, suggest that poor baseline performance subjects tended to gain more after training, while the learning rates were much less affected by the baseline performance. In particular, the training gains for *d’* (verbal 3-back: *p* < 0.01, *r*^2^ = 0.58, *R* = −0.76. shape 3-back: *p* = 0.18, *r*^2^ = 0.06, *R* = −0.25), ACC (verbal 3-back: *p* < 0.01, *r*^2^ = 0.49, *R* = −0.70. shape 3-back: *p* < 0.05, *r*^2^ = 0.23, *R* = −0.48) were significantly correlated with the corresponding baseline performance in both verbal 3-back and shape 3-back tasks. High baseline RT subjects also tended to get large reductions of RT in verbal 3-back (*p* < 0.05, *r*^2^ = 0.21, *R* = −0.46) but not in shape 3-back (*p* = 0.79, *r*^2^ = 0.00, *R* = −0.05) tasks. Turning to the learning rates, no significant linear relationship was found for the learning rates of *d’* (verbal 3-back: *p* = 0.81, *r*^2^ = 0.00, *R* = −0.05. verbal 4-back: *p* = 0.90, *r*^2^ = 0.00, *R* = 0.02) and ACC (verbal 3-back: *p* = 0.29, *r*^2^ = 0.04, *R* = 0.20. verbal 4-back: *p* = 0.06, *r*^2^ = 0.12, *R* = −0.35) in both verbal 3- and 4-back tasks. For learning rates of RT, high baseline RT subjects tended to have high learning rates in verbal 3-back (*p* < 0.01, *r*^2^ = 0.34, *R* = −0.59) but not in verbal 4-back tasks (*p* = 0.78, *r*^2^ = 0.00, *R* = −0.05).

The above results suggest that the training effects were affected by the baseline performance of the subjects, especially performance changes. Therefore, the baseline performance metrics were compared between the active and sham group, with bootstrapping based *T*-tests. No significant difference was found for the baseline *d’* (verbal 3-back: *q*_(FDR)_ > 0.05, *t*_(28)_ = −1.19, shape 3-back: *q*_(FDR)_ > 0.05, *t*_(28)_ = −0.16), response accuracy (verbal 3-back: *q*_(FDR)_ > 0.05, *t*_(28)_ = −0.03, shape 3-back: *q*_(FDR)_ > 0.05, *t*_(28)_ = 0.06) and RT (verbal 3-back: *q*_(FDR)_ > 0.05, *t*_(28)_ = −0.97, shape 3-back: *q*_(FDR)_ > 0.05, *t*_(28)_ = −1.30). The learning rates and training gains of the performance metrics were then divided into a high baseline performance group (HBPG) and a low baseline performance group (LBPG) according to pre-training performance. The medians of the baseline performance metrics served as the threshold, in order to group the data, and were excluded from further analyses. Then, comparisons between HBPG and LBPG were performed for the active and shame group, respectively. Significant differences were found only for Δ *d*’ of the active group in verbal 3-back (*q*_(FDR)_ < 0.01, *t*_(12)_ = 4.15, power = 0.83) and Δ ACC of the active group (*q*_(FDR)_ < 0.05, *t*_(12)_ = 3.18, power = 0.74) and sham group (*q*_(FDR)_ < 0.05, *t*_(12)_ = 2.73, power = 0.68) in verbal 3-back. No other significant differences were found.

## Discussion and Conclusion

This study examined the effect of anodal HD-tDCS on the variation of performance during load-increasing WM training. HD-tDCS was paired with a five-session WM training task. The results showed that learning rates and training gains of the performance metrics, benefited from anodal HD-tDCS of left DLPFC. The advantage induced by tDCS could be transferred to a similar untrained WM task. Further analysis found that the training gains, rather than the learning rates, tended to negatively correlate with baseline performance. These results show the promise of utilizing tDCS as an intervention to augment WM training and to improve WM-related skills for those in need, especially those with poor cognitive performance.

In the previous studies on learning rates, the learning curves were obtained from the highest load factor the participants could reach in each session, like the final span of WM span task (Richmond et al., 2014) and the largest n of the n-back task (Ruf et al., 2017) from daily training sessions. In this study, we first explored the performance variation of the stable-load task across the load-adaptive training process, through performing verbal 3-back and 4-back tasks at the beginning of each training session. The results showed a steeper learning curve of the load factor for the active group. Moreover, the differences in learning rates of *d’* and ACC in both verbal 3-back and verbal 4-back tasks, reached significance when compared between the active and sham group. It is worth emphasizing that only *d’* and ACC of the active group tended to increase with the number of training sessions. The results of the statistical power analyses also showed a high power of the significance. These results showed the promising benefits of tDCS on WM training, by not only enlarging memory capacity, but also enhancing the performance of stable-load tasks.

As for the training outcomes, the results showed distinct trends that the gains of the active group benefited more from training, than those of the sham group. Moreover, this kind of advantage could be transferred from a trained task to a similar untrained task. The statistical powers however, were relatively low. Further analysis revealed a significant negative correlation between the training gains and the baseline performance, whereas only the learning rates of RTs were found to be negatively correlated with the baseline RTs. Therefore, participants with lower baseline performance tended to benefit more from WM training. The efficacy of tDCS has been found to be regulated by some basal conditions in previous studies (Hill et al., 2016a). Distinct effects of tDCS on behavior outcomes in healthy and depressed participants have also been found in an emotional WM task (Moreno et al., 2015). For healthy cohorts, baseline performance may also regulate the behavioral consequences. A study conducted by Gözenman and Berryhill found that tDCS produced greater beneficial outcomes for low a WM capacity group than for a high WM capacity group (Gözenman and Berryhill, 2016). These findings suggest that tDCS may potentially produce considerable behavioral benefits for both healthy people and those with neuropsychiatric disorders, and that the beneficial consequences tend to depend on the behavioral and neurophysiological baseline state of the target demographic (Moreno et al., 2015; Gözenman and Berryhill, 2016; Hill et al., 2016a; Talsma et al., 2017). These studies also confirmed that baseline conditions of participants should be considered in future studies.

When repeated sessions of tDCS are used, ethical and safety concerns should be adequately addressed. In fact, repeated-session protocol has been widely used in both healthy and neuropsychiatric cohorts in the studies reviewed in the Introduction section (Richmond et al., 2014; Jones et al., 2015; Au et al., 2016; Stephens and Berryhill, 2016; Katz et al., 2017; Krause et al., 2017; Ruf et al., 2017; dos Santos et al., 2018). By reviewing hundreds of studies that involved thousands of healthy or neuropsychiatric participants, several recent publications have discussed the question on ethics and safety and found that repeated sessions of active tDCS did not increase the risk (serious adverse effect or irreversible injury) to participants, compared to the sham tDCS (Fertonani et al., 2015; Bikson et al., 2016; Matsumoto and Ugawa, 2017; Nikolin et al., 2018). The stimulation parameters in this study have been configured according to the conclusions of these publications and approved by the ethics committee. The participants were fully informed of the possible consequences of tDCS before they agreed to participate in this study. No physical or mental abnormality was reported during or after the stimulation, except for a slight tingling sensation under the electrode.

The current study was mainly concerned with participants’ behavioral stable-load tasks results, during tDCS-paired load-adaptive WM training procedures. The underlying neural mechanism of the advantages induced by tDCS was not explored in this study. Future research will further explore the variations of neural responses and their relationship with the behavioral outcomes during tDCS-paired cognitive training, especially the effect of baseline neuronal properties on the efficacy of tDCS-paired training. The neuroimaging techniques like EEG (Sood et al., 2016), fNIRS (Khan et al., 2013; McKendrick et al., 2015), functional magnetic resonance imaging (fMRI; Callan et al., 2016) and the combination of transcranial magnetic stimulation with EEG (TMS-EEG; Hill et al., 2016b) are promising options to explore the mechanisms of tDCS and to further improve tDCS application in future research.

In summary, the current study provides evidence that anodal HD-tDCS of the left DLPFC has the potential to enhance WM training, by facilitating the training process and producing more training improvements. Moreover, the advantage of training gains induced by tDCS, has the potential to be transferred to the similar untrained task. These findings endorse the notion that tDCS-paired multi-session WM training can be leveraged as a tool to augment the ability in WM-intensive tasks.

## Author Contributions

YK: conceptualization, planning, data collection, data analysis, and writing of the manuscript. NW: conceptualization, data collection, and proofreading. JD: data collection and supporting data analysis. LK: data collection and proofreading. SL: supporting data collection. MX: conceptualization and supporting data analysis. XA: conceptualization and supporting data analysis. DM: conceptualization, planning, supporting data analysis and proofreading.

## Conflict of Interest Statement

The authors declare that the research was conducted in the absence of any commercial or financial relationships that could be construed as a potential conflict of interest.

## Footnotes

^1^https://webpower.psychstat.org/wiki/
